# Supplementary material for: Role of tankyrase scaffolding in the β-catenin destruction complex and WNT signaling
Source: bioRxiv. 2025 Sep 24:2025.09.22.677768. Preprint. [Version 1] doi: 10.1101/2025.09.22.677768 (PMC12485674; doi:10.1101/2025.09.22.677768)
Supplement: Supplement 1 [file NIHPP2025.09.22.677768v1-supplement-1.pdf]

bioRxiv preprint doi: https://doi.org/10.1101/2025.09.22.677768; this version posted September 24, 2025. The copyright holder for this preprint (which was not certified by peer review) is the author/funder, who has granted bioRxiv a license to display the preprint in perpetuity. It is made available under aCC-BY 4.0 International license.

Supplementary Figures

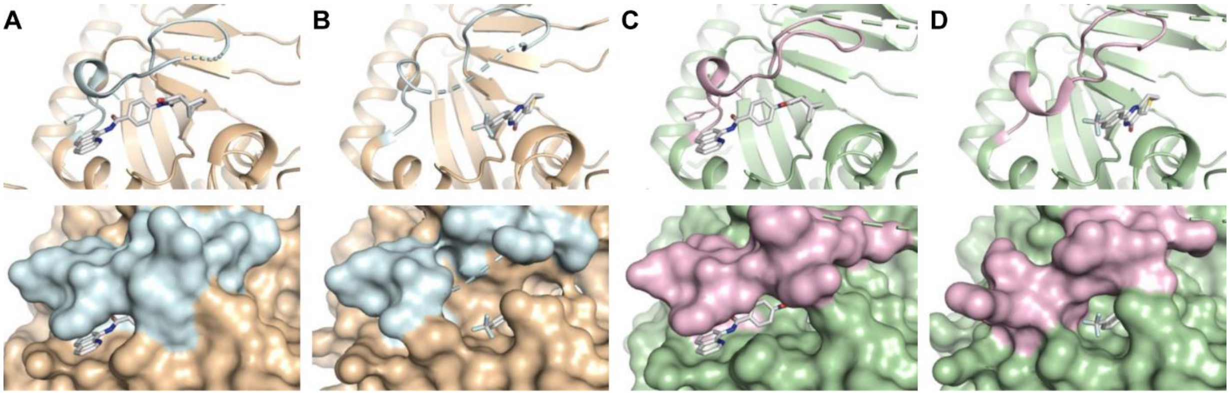

Figure S1. Crystal structures used to guide the design of PROTAC molecules

(A) The crystal structure of TNKS1 with IWR1-exo (PDB 4OA7).

(B) The crystal structure of TNKS1 with XAV939 (PDB 3UH4).

(C) The crystal structure of TNKS2 with IWR1 (PDB 3UA9).

(D) The crystal structure of TNKS2 with XAV939 (PDB 3KR8).

bioRxiv preprint doi: https://doi.org/10.1101/2025.09.22.677768; this version posted September 24, 2025. The copyright holder for this preprint (which was not certified by peer review) is the author/funder, who has granted bioRxiv a license to display the preprint in perpetuity. It is made available under aCC-BY 4.0 International license.

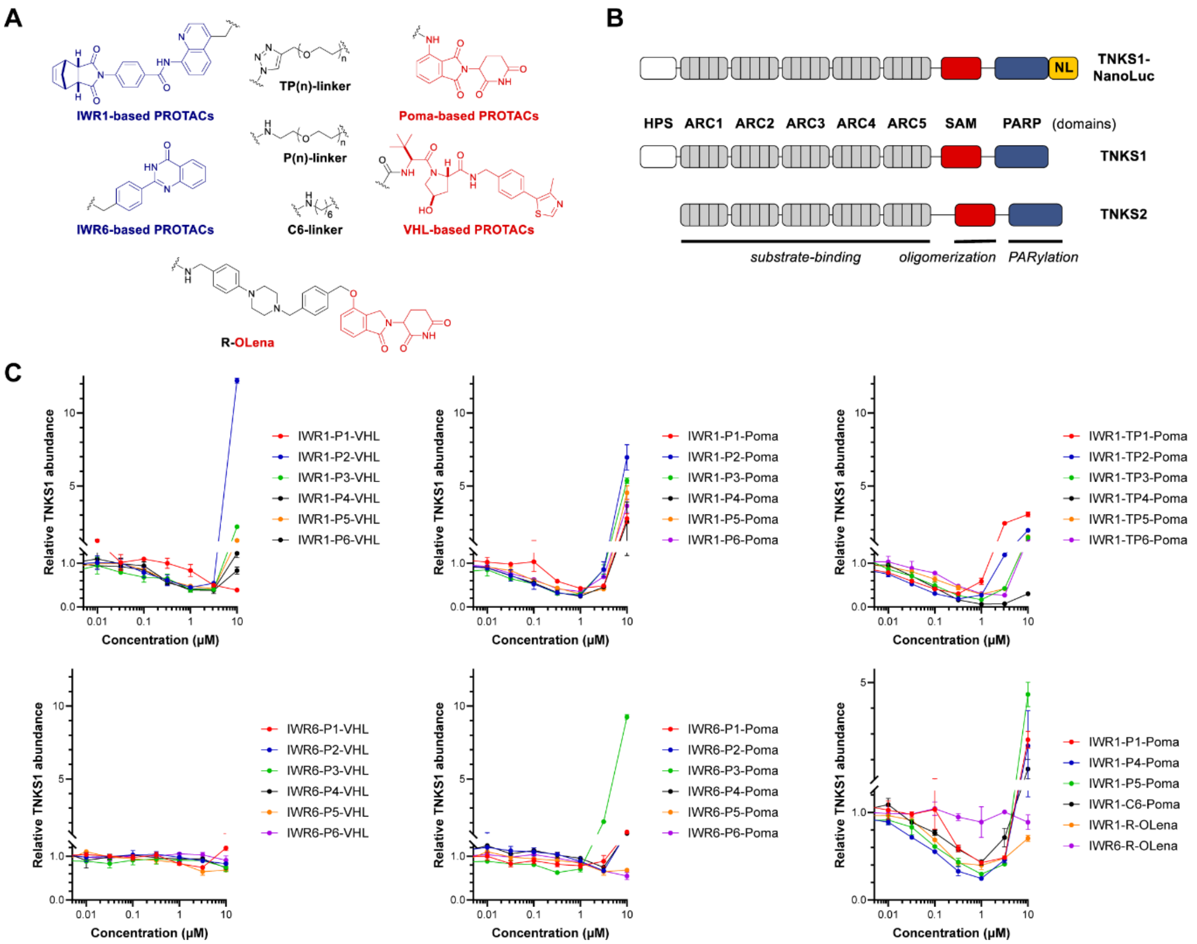

Figure S2. Identification of active PROTAC molecules using CRISPR engineered HAP1 cells expressing a TNKS1-NanoLuc fusion protein

(A) The chemical structures of the PROTAC molecules.

(B) The schematic diagrams of the domain structures of TNKS1, TNKS2 and TNKS1-NanoLuc.

(C) The relative abundance of the endogenous TNKS1 was measured by the luciferase activity upon treating HAP1-TNKS1-NanoLuc cells with IWR1-P(n)-VHL, IWR6-P(n)-VHL, IWR1-P(n)-Poma, IWR6-P(n)-Poma, or IWR1-TP(n)-Poma. IWR1-R-Olena bearing a rigid linker of length and polarity comparable to IWR1-P4-Poma and IWR1-P5-Poma alleviated the hook effect but was less effectively in promoting TNKS1 degradation. Removing the oxygen atom from the linker of IWR1-P1-Poma gave IWR1-C6-Poma with a more hydrophobic linker, but there was no improvement in the degradation efficacy.

bioRxiv preprint doi: https://doi.org/10.1101/2025.09.22.677768; this version posted September 24, 2025. The copyright holder for this preprint (which was not certified by peer review) is the author/funder, who has granted bioRxiv a license to display the preprint in perpetuity. It is made available under aCC-BY 4.0 International license.

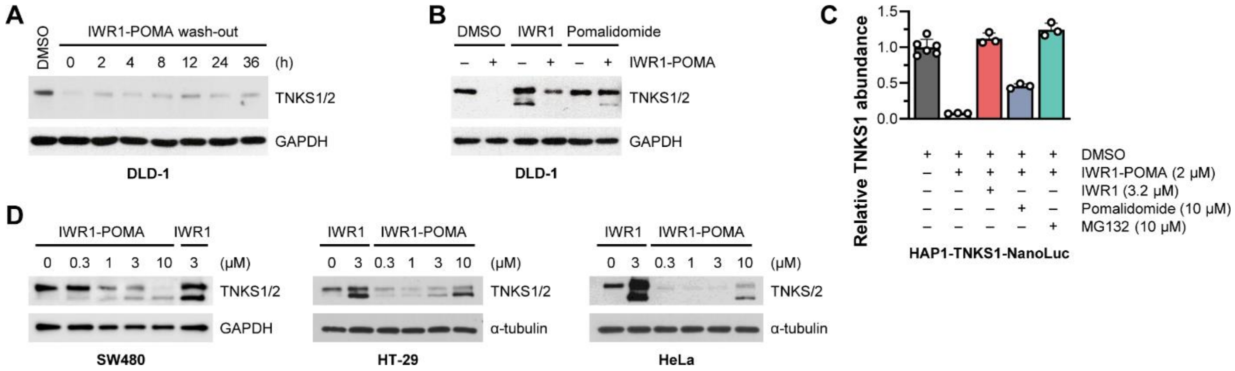

Figure S3. Additional characterization of IWR1-POMA

(A) TNKS did not recover in DLD-1 cells at least 36 h after removing IWR1-POMA.

(B) IWR1 (3 μM) and pomalidomide (3 μM) prevented the degradation of TNKS by IWR1-POMA (3 μM) in DLD-1 cells.

(C) IWR1, pomalidomide, and MG132 blocked the degradation of TNKS by IWR1-POMA in HAP1-TNKS-NanoLuc cells.

(D) IWR1-POMA promoted TNKS degradation while IWR1 induced TNKS accumulation in SW480, HT-29 and HeLa cells.

bioRxiv preprint doi: https://doi.org/10.1101/2025.09.22.677768; this version posted September 24, 2025. The copyright holder for this preprint (which was not certified by peer review) is the author/funder, who has granted bioRxiv a license to display the preprint in perpetuity. It is made available under aCC-BY 4.0 International license.

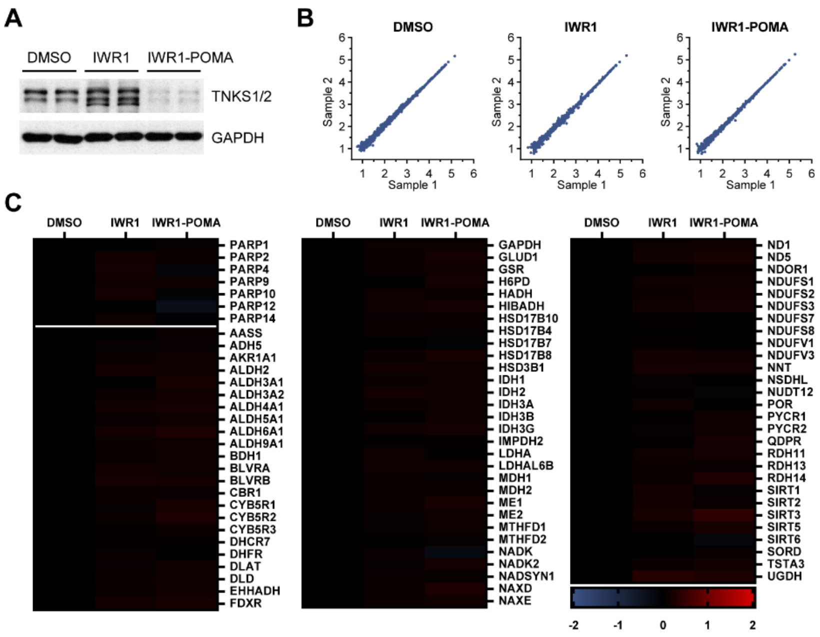

Figure S4. Proteomic analysis of DLD-1 cells treated with DMSO, IWR1 or IWR1-POMA

(A) Western blot analysis of samples corresponding to Figure 1 E and 1F confirmed the accumulation of TNKS1/2 by IWR1 and the depletion by IWR1-POMA.

(B) Correlation analysis showed high reproducibility between the two biological repeats.

(C) IWR1-POMA selectively degraded TNKS without inducing appreciable perturbations to 7 other PARP family member proteins and 79 NAD(P)-dependent enzymes detected in this proteomic experiment.

bioRxiv preprint doi: https://doi.org/10.1101/2025.09.22.677768; this version posted September 24, 2025. The copyright holder for this preprint (which was not certified by peer review) is the author/funder, who has granted bioRxiv a license to display the preprint in perpetuity. It is made available under aCC-BY 4.0 International license.

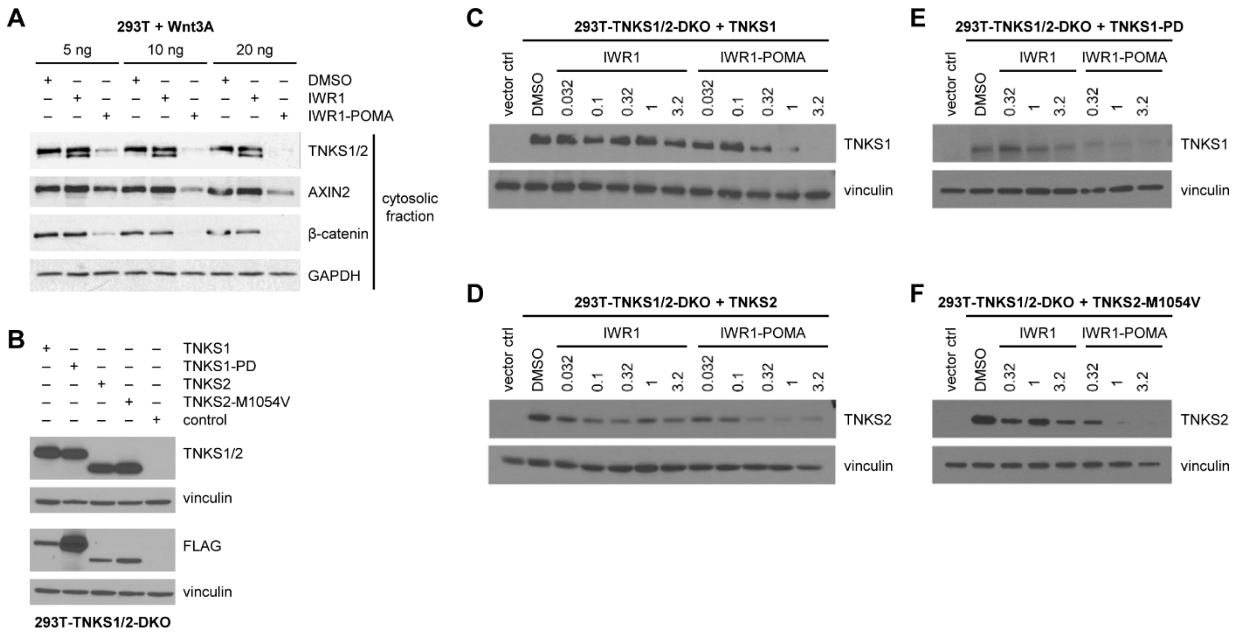

Figure S5. Degradation of TNKS by IWR1-POMA in 293T cells

(A) 293T cells were transfected with different doses of Wnt3A plasmid and then treated with DMSO, IWR1 (3 μM) or IWR1-POMA (3 μM). The cytosolic fraction of the cell lysates was then examined by Western blot. IWR1-POMA promoted a more complete degradation of β-catenin than IWR1 and reduced the level of the WNT target AXIN2.

(B) Western blot analysis confirmed the lack of TNKS expression in TNKS1/2-DKO cells and validated the expression of FLAG-TNKS1, 3×FLAG-TNKS1-PD, FLAG-TNKS2 and FLAG-TNKS2-M1054V after transfection.

(C) Western blot analysis of samples corresponding to Figure 2A confirmed the degradation of TNKS1 by IWR1-POMA.

(D) Western blot analysis of samples corresponding to Figure 2B confirmed the degradation of TNKS2 by IWR1-POMA.

(E) Western blot analysis of samples corresponding to Figure 2C confirmed the degradation of TNKS1-PD by IWR1-POMA.

(F) Western blot analysis of samples corresponding to Figure 2D confirmed the degradation of TNKS2-M1054V by IWR1-POMA.

bioRxiv preprint doi: https://doi.org/10.1101/2025.09.22.677768; this version posted September 24, 2025. The copyright holder for this preprint (which was not certified by peer review) is the author/funder, who has granted bioRxiv a license to display the preprint in perpetuity. It is made available under aCC-BY 4.0 International license.

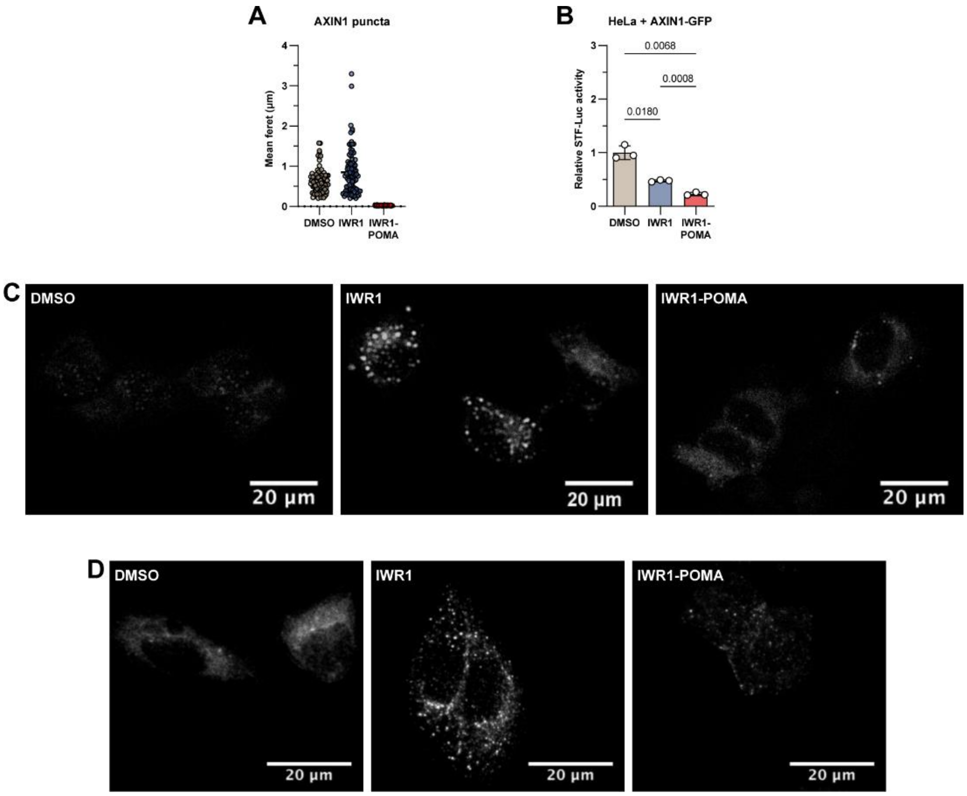

Figure S6. TNKSi induced the formation of AXIN puncta

(A) Puncta count of samples corresponding to Figure 4C.

(B) HeLa cells transfected with AXIN1-GFP and STF plasmids and then treated with DMSO, IWR1 (3 μM) or IWR1-POMA (3 μM). IWR1-POMA suppressed WNT/β-catenin signaling significantly better than IWR1, indicating that AXIN puncta formation is not required for the DC to promote β-catenin degradation. The data is presented as mean ± SEM with p-values calculated by two-tailed unpaired t-test.

(C) HeLa cells transfected with GFP-AXIN1 followed by treating with DMSO, IWR1 (3 μM) or IWR1-POMA (3 μM). Together with Figure 4C, this experiment shows that the position of GFP tag does not affect puncta formation.

(D) HeLa cells transfected with GFP-AXIN2 followed by treating with DMSO, IWR1 (3 μM) or IWR1-POMA (3 μM). This experiment shows that AXIN2 also forms puncta upon IWR1 treatment.

bioRxiv preprint doi: https://doi.org/10.1101/2025.09.22.677768; this version posted September 24, 2025. The copyright holder for this preprint (which was not certified by peer review) is the author/funder, who has granted bioRxiv a license to display the preprint in perpetuity. It is made available under aCC-BY 4.0 International license.

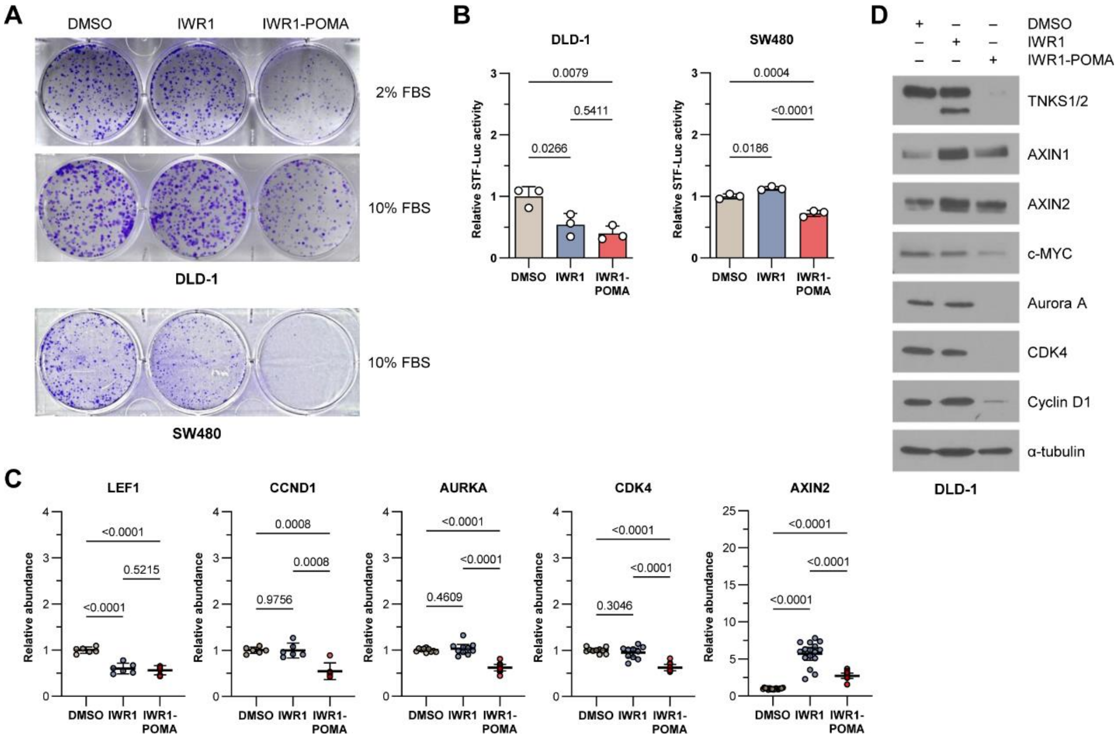

Figure S7. IWR1-POMA suppressed CRC growth through WNT inhibition

(A) IWR1-POMA (3 μM) suppressed DLD-1 and SW480 colony formation.

(B) IWR1-POMA (3 μM) suppressed of WNT signaling more effectively than IWR1 (3 μM) in DLD-1 and SW480 cells. The data is presented as mean ± SEM with p-values calculated by two-tailed unpaired t-test.

(C) Peptide abundance of WNT targets, corresponding to Figure 1E and 1F. IWR1-POMA (3 μM) controlled several WNT targets not regulated by IWR1 (3 μM) in DLD-1 cells.

(D) Western blot analysis confirmed that c-MYC, Aurora A, CDK4 and cyclin D1 responded to IWR1-POMA (3 μM) but not IWR1 (3 μM) in DLD-1 cells.

bioRxiv preprint doi: https://doi.org/10.1101/2025.09.22.677768; this version posted September 24, 2025. The copyright holder for this preprint (which was not certified by peer review) is the author/funder, who has granted bioRxiv a license to display the preprint in perpetuity. It is made available under aCC-BY 4.0 International license.

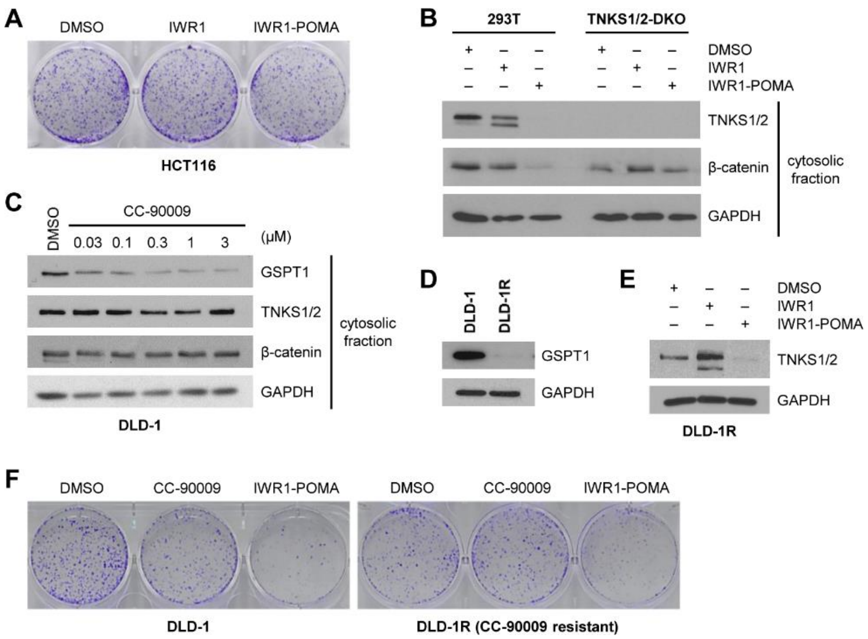

Figure S8. IWR1-POMA suppressed CRC proliferation through on-target WNT inhibition

(A) HCT116 cells carrying a mutation in β-catenin that could not be processed by the DC were resistant to both IWR1 (3 μM) and IWR1-POMA (3 μM).

(B) IWR1-POMA (3 μM) reduced the cytosolic β-catenin level more effectively than IWR1 (3 μM) in 293T cells. The level of cytosolic β-catenin in 293T-TNKS1/2-DKO cells that lack both TNKS1 and TNKS2 did not change with either drug treatment.

(C) CC-90009 induced GSPT1 degradation in DLD-1 cells but had no effect on TNKS. GSPT2 was not detectable by Western blot, which is consistent with the reported GSPT levels determined by quantitative proteomic analysis in this cell line—102,567 ppb for GSPT1 and 2,495 ppb for GSPT2 (https://www.ebi.ac.uk/gxa/experiments/E-PROT-18/Results)[52].

(D) DLD-1R cells obtained from cultivating DLD-1 cells with CC-90009 have a dramatically reduced level of GSPT1 expression meanwhile maintaining normal TNKS expression.

(E) IWR1 (3 μM) promoted TNKS accumulation and IWR1-POMA (3 μM) induced TNKS degradation in DLD-1R cells.

(F) IWR1-POMA prevented colony formation of DLD-1R cells deficient in GSPT1/2.

bioRxiv preprint doi: https://doi.org/10.1101/2025.09.22.677768; this version posted September 24, 2025. The copyright holder for this preprint (which was not certified by peer review) is the author/funder, who has granted bioRxiv a license to display the preprint in perpetuity. It is made available under aCC-BY 4.0 International license.

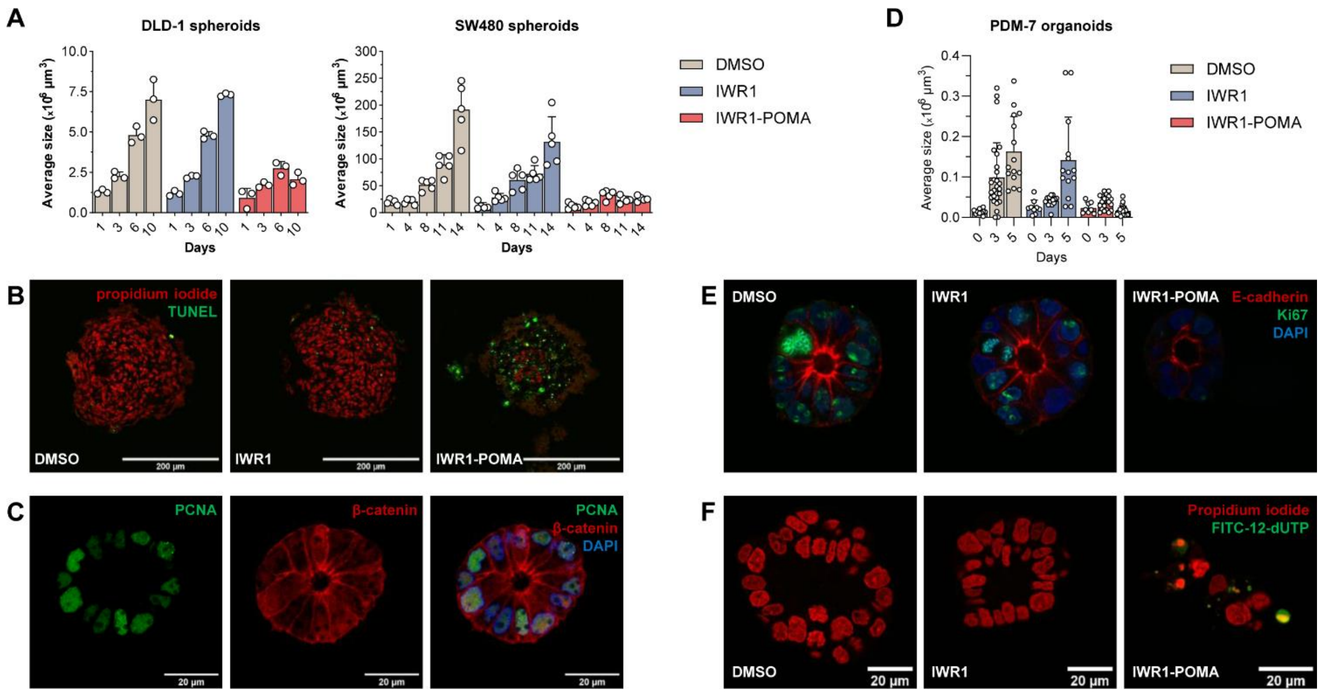

Figure S9. IWR1-POMA demonstrated efficacy in CRC spheroid and primary organoid models

(A) The growth chart of DLD-1 and SW480 spheroids treated with DMSO, IWR1 (5 μM), or IWR1-POMA (5 μM). The sizes represent the apparent dimensions of the spheroids including the peripheral dead cells.

(B) IWR1-POMA (5 μM) induced apoptosis in DLD-1 spheroids.

(C) PDM-7 organoids grown from single cells preserved the heterogeneous nature of CRC tumors.

(D) IWR1-POMA (1 μM) prevented the formation of PDM-7 organoids from single cells while IWR1 (1 μM) did not.

(E and F) IWR1-POMA (1 μM) suppressed proliferation and induced apoptosis in PDM-7 organoids grown from single cells while IWR1 (1 μM) had little effect.
